# One Direction? A Tutorial for Circular Data Analysis Using R With Examples in Cognitive Psychology

**DOI:** 10.3389/fpsyg.2018.02040

**Published:** 2018-10-30

**Authors:** Jolien Cremers, Irene Klugkist

**Affiliations:** Department of Methodology and Statistics, Faculty of Social and Behavioural Sciences, Utrecht University, Utrecht, Netherlands

**Keywords:** tutorial, circular data, general linear models, mixed-effects models, projected normal distribution

## Abstract

Circular data is data that is measured on a circle in degrees or radians. It is fundamentally different from linear data due to its periodic nature (0° = 360°). Circular data arises in a large variety of research fields. Among others in ecology, the medical sciences, personality measurement, educational science, sociology, and political science circular data is collected. The most direct examples of circular data within the social sciences arise in cognitive and experimental psychology. However, despite numerous examples of circular data being collected in different areas of cognitive and experimental psychology, the knowledge of this type of data is not well-spread and literature in which these types of data are analyzed using methods for circular data is relatively scarce. This paper therefore aims to give a tutorial in working with and analyzing circular data to researchers in cognitive psychology and the social sciences in general. It will do so by focusing on data inspection, model fit, estimation and hypothesis testing for two specific models for circular data using packages from the statistical programming language R.

## 1. Introduction

Circular data arises in almost all fields of research, from ecology where data on the movement direction of animals is investigated (Rivest et al., 2015) to the medical sciences where protein structure (Mardia et al., 2006) or neuronal activity (Rutishauser et al., 2010) is investigated using periodic and thus circular measurements. The most direct examples of circular data within the social sciences arise in cognitive and experimental psychology. For example, in experiments on cognitive maps the human sense of direction is investigated through asking participants in a study to point north (Brunyé et al., 2015) or to walk to a target object (Warren et al., 2017). The closer the participants' pointing or walking direction was to the actual north or target object, the better their sense of direction. Other examples include the visual perception of space (Matsushima et al., 2014), visual working memory (Heyes et al., 2016) and sensorimotor synchronization in music making (Kirschner and Tomasello, 2009).

However, despite the fact that circular data is being collected in different areas of cognitive and experimental psychology, the knowledge of this type of data is not well-spread. Circular data is fundamentally different from linear data due to its periodic nature. On the circle, measurements at 0° and 360° represent the same direction whereas on a linear scale they would be located at opposite ends of a scale. For this reason circular data require specific analysis methods. Some less technical textbooks on analysis methods for circular data have been written (Batschelet, 1981; Fisher, 1995; Pewsey et al., 2013). However, these works are not part of the “standard” texts on statistical analysis in psychology or the social sciences in general nor are they very well known amongst social scientific researchers.

Therefore, this paper aims at giving a tutorial in working with and analysing circular data to researchers in cognitive psychology and the social sciences in general. The main goal of this tutorial is to explain how to inspect and analyse your data when the outcome variable is circular. We will discuss data inspection, model fit, estimation and hypothesis testing in general linear models (GLM) and mixed-effects models. In this tutorial we decide to mainly focus on one particular approach to the analysis of circular data, the embedding approach. We do so for the flexibility of this approach and the resulting variety in types of models that have already been outlined in the literature on circular data for this approach. Note that for an optimal understanding of the paper, the reader should ideally have some knowledge on R (R Core Team, 2017) and on GLM and mixed-effects models in the linear setting. The reader does not need to be familiar with circular data.

The structure of the tutorial is such that the reader is guided by two examples throughout the paper. One is an example for an ANOVA model and the other for a mixed-effects model. First however, we give a short introduction to circular data in general. Then we introduce the ANOVA example after which descriptive methods for circular data are explained through a section on data inspection for this example. After that we will continue with an analysis of the example datasets. First we analyse the ANOVA dataset using a method for circular GLM and give interpretation guidelines for this model. Subsequently we will introduce and analyse the mixed-effects example data. Again, we analyse this data and include guidelines for interpretation. The analyses of both datasets, the ANOVA and mixed-effects dataset, are performed using the R package bpnreg (Cremers, 2018). For both models, the GLM and the mixed-effects model for a circular outcome, we write a short technical section in which the mathematical details of the respective models are given. Lastly, we give a summary of the paper and additional references to literature on other models for circular data in the concluding remarks.

## 2. Circular data

**Figure 1:**
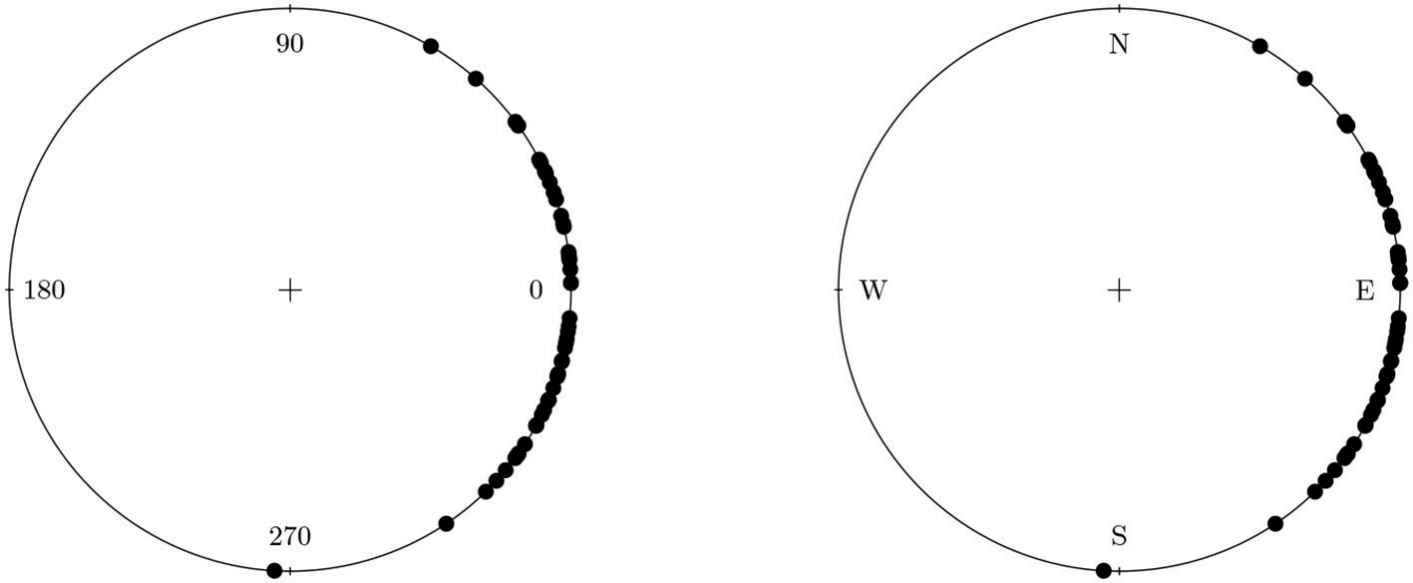
Data from participants in an experiment that were instructed to move East. The plot on the left shows the data on a 0° −360° scale. The plot on the right shows the data on the compass.

In the introduction we have briefly mentioned that circular data is data of a periodic nature. The most intuitive form of circular data comes in the form of directions on a compass. For example, a participant in an experiment could be instructed to move or point to a certain target. We can then measure the direction, North, South, East or West on a scale from 0 to 360°. A plot with simulated data containing such measurements for several participants is shown in Figure 1. In this plot we can easily see the periodicity of the data, 0° represents the same datapoint as 360°. Furthermore, we can see what happens if we would treat this data in the “usual” linear way. Participants that moved North-East have a score of 45° and participants that moves South-East have a score of 315°. On the circle we can see that this is only 90° apart, while on a linear scale it is much further apart at 315°–45° = 270°. More importantly however, there is a difference between the circular and linear means for this data. In Figure 1 we see that the circular mean direction is 0°. The linear mean however is 180° and is opposite to the actual mean direction of the data. Clearly, a linear treatment of the data in Figure 1 can lead to incorrect conclusions.

**Figure 2:**
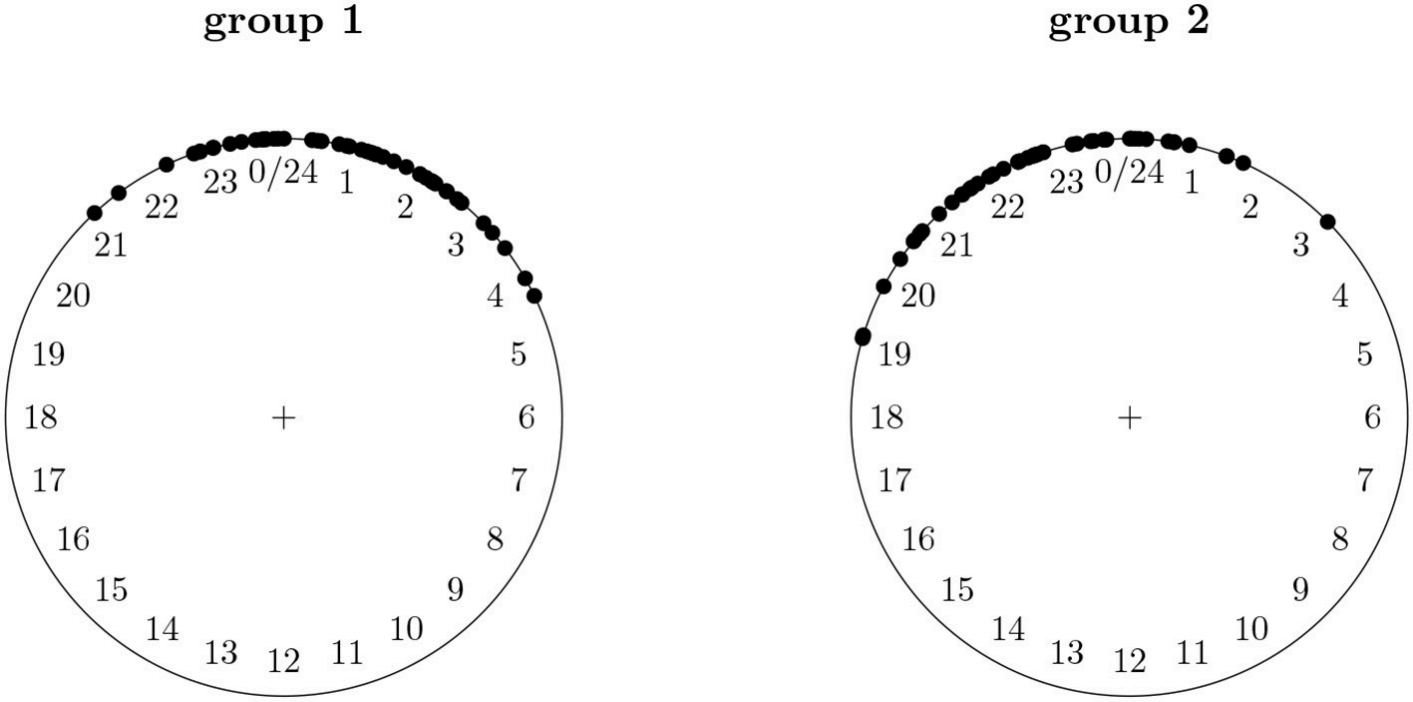
Data for the hour at which positive affect is highest for two groups of psychiatrical patients who are being treated for depression at different clinics.

Clock times are another type of circular data. We might for instance be interested at what time of day a certain event takes place, e.g., the time of day at which positive affect is highest. Figure 2 shows simulated data for the time of day at which positive affect is highest for two groups of participants, e.g., two groups of psychiatrical patients who are being treated for depression at different clinics. From the plots we clearly see that the peak of positive affect for the two groups is at roughly the same time of day, one slightly before 12 p.m. and one slighly after. However, if we were to analyze this data using standard statistics for linear data and we would compare the means of the two groups, 11 p.m. and 1 a.m. we would reach a completely different conclusion. The two means are namely at the two opposite ends of a linear scale from 00.00 a.m. to 12.00 p.m., and we would conclude that the time of day at which positive affect is highest is different for the two groups.

The two examples of circular data that we have just given illustrate why it is important to treat circular data differently from linear data. This goes both for describing your data, e.g., computing circular means, as well as analyzing them, e.g., testing whether the circular means of two groups differ. In the next section we will introduce an example dataset on which we will show several ways to inspect and compute descriptive measures for circular data.

## 3. Inspecting your data

In the previous section we have seen that the computation of a circular mean differs from that of a linear mean. Methods for data inspection, the computation of descriptive statistics and plotting methods, are different for circular data. Because data inspection shoud be done before performing inference of any kind we will outline a basic way to inspect circular data using the R packages bpnreg (Cremers, 2018) and circular (Agostinelli and Lund, 2017). We will discuss plots and several descriptive measures for circular data using an example dataset, the motor resonance data.

### 3.1. The motor resonance data

In this section we introduce data from an article by Puglisi et al. (2017) on human motor resonance. From now on we will call this data the motor resonance data. Motor resonance is a response in the brain in the primary motor cortex and spinal circuits that is caused by observation of others' actions. In their research Puglisi et al. (2017) conduct an experiment in which “observers” are asked to either look at the movement of a hand of a “mover” or at another object in order to evaluate the role of attention in motor resonant response. The experiment has three conditions: the “explicit observation” condition (*n* = 14), where observers are explicitly instructed to observe the hand, the “semi-implicit observation” condition (*n* = 14) where the observers have to perform a task that requires the implicit observation of the hand of the mover and the “implicit observation” condition (*n* = 14), where observers have to perform a task that is independent of the observation of the hand of the mover. The idea of motor resonance is that the “observer” starts moving his or her hand in the same manner as the “mover” because he or she is implicitly or explicitly observing the hand of the “mover.” This is the resonant response. This resonant response is hypothesized to be strongest and more synchronized with the hand movement of the mover in the explicit condition and smallest in the implicit condition. In each condition the hand movements of the observers were measured and the phase difference between movement of the observers' hand and the hand of the mover was calculated. A phase difference can be expressed in degrees or time and is formally defined as the difference at a specific point in time between two waves that have the same frequency. In the motor resonance data the phase difference is a measurement of the strength of the resonant response and a circular variable. It can thus be described and analyzed using circular statistics. In addition to the phase difference the average amplitute of the hand movement of the observer was computed. Note that in the original article there was also a baseline condition (*n* = 14) without a mover. In this condition, observers had to look at an inanimate object that moves in an identical manner to the hand of the mover in other conditions. The baseline condition is however not included in the example data since no resonant response was observerd in the observers' hand according to the original research.

The motor resonance data can be found in the package bpnreg as the dataframe Motor. Motor is a dataframe with 42 rows and seven variables. The variable Cond indicates the condition (explicit, semi-implicit and implicit) a participant was placed in, the AvAmp variable contains the average amplitude, and the PhaseDiff and Phaserad variables contain the measured phase difference between “observer” and “mover” in degrees and radians, respectively. Note that circular data can be represented both in degrees on a scale from 0° to 360° and in radians on a scale from 0 to 2π (1 degree = 1 * π/180° radians).

### 3.2. Plots for circular data

The main question of interest for the motor resonance data is whether the phase difference between the three experimental conditions differs. To be more precise whether there is a smaller phase difference in the explicit condition than in the other two. Differences that are observed in the phase difference are interpreted as differences in the strength of the resonant response (Puglisi et al., 2017). A smaller phase difference indicates a stronger and more synchronized resonant response. A first step to investigating the question of interest is plotting the phase differences of the three conditions. We can do so using the package circular.

**Figure 3:**
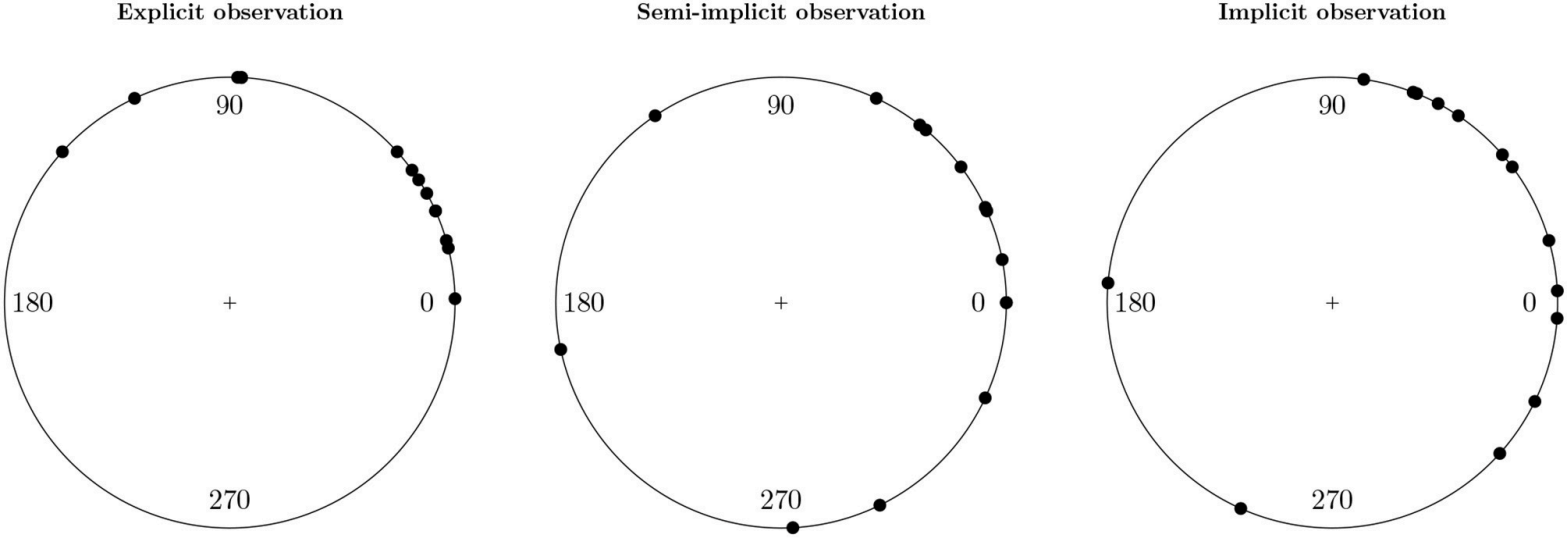
Plots of the phase differences for each condition of the motor resonance data.

Figure 3 shows the plots of the phase differences of each condition. We see that the phase differences in the explicit condition are much less spread out on the circle than the phase differences in the other two conditions. Also the average phase differences seem to differ between the conditions. In the next section we show measures for the mean and variance of a sample of circular data.

### 3.3. Circular mean, resultant length, and variance

Table 1 shows descriptives for the motor resonance data. For each group, the table contains sample statistics for the circular mean and mean resultant length of the phase difference. The circular population mean, μ, indicates the average direction of a certain variable in the population. The population mean resultant length, ρ, is a statistic between 0 and 1 that gives us information on the spread of a circular variable in the population. It is interpreted as a precision measure where 0 means that the spread is large and 1 means that all data are concentrated at a single value. Sample statistics for these values are $\bar{\theta }$ for the mean and $\bar{R}$ for the mean resultant length.

**Table 1:** Descriptives for the motor resonance data with mean direction ($\bar{\theta }$), mean resultant length ($\bar{R}$), circular variance (*V*_*m*_) and circular standard deviation (*v*) of the phase difference for each condition.

**Phase difference**	**$\bar{\theta }$**	**$\bar{R}$**	***V*_*m*_**	***v***
Explicit	49.55°	0.77	0.23	41.39°
Semi.implicit	18.45°	0.54	0.46	63.82°
Implicit	31.94°	0.56	0.44	61.72°

**Figure 4:**
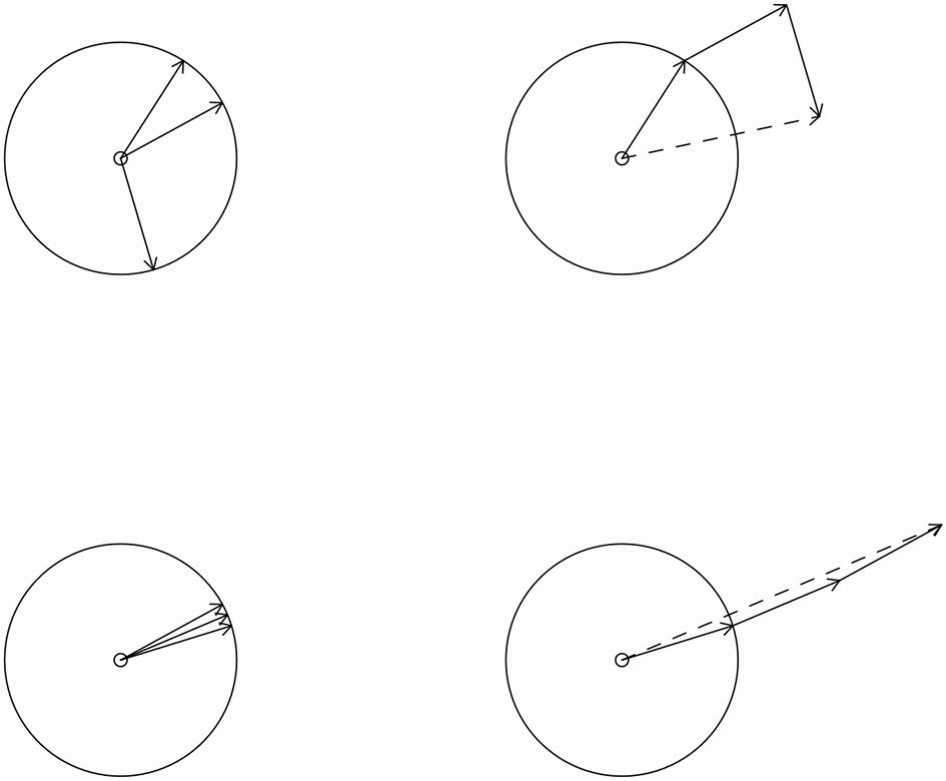
The computation of a circular mean and (mean) resultant length **(right)** for two sets of circular data **(left)**. The solid lines are vectors representing the circular datapoints. The direction of the dotted vector is the mean direction and the length of the dotted vector is the resultant length.

Graphically we can illustrate the computation of $\bar{\theta }$ and $\bar{R}$ as shown in Figure 4. On the left side of this figure we see two sets of circular data. We represent a circular datapoint as a vector composed of the cosine and sine of the datapoint instead of one value measured in degrees (or radians). Example for a score of 90° we have the following vector (cos(90°), sin(90°)). The solid vectors in Figure 4 each represent one circular datapoint. To compute the mean directions and resultant lengths for the datasets on the left we place the vectors head to toe, as in the right side of Figure 4. We then connect the toe of the first vector to the head of the last vector. This results in the dotted vectors on the right side of Figure 4. The direction of the dotted vector is the mean direction, $\bar{\theta }$ of the vectors from which it was created. The length of the dotted vector is the resultant length. The mean resultant length, $\bar{R}$ is the length of this vector divided by the number of vectors from which it was created. In Figure 4 we see that the data in the bottom left figure are much more concentrated on the circle than the data in the upper left figure. This translates to the resultant length in the bottom right being larger than the resultant length (length of the dotted vector) in the upper right. Formulae for the computation of $\bar{\theta }$ and $\bar{R}$ can be found in Fisher (1995).

In Table 1 we see that in the motor resonance data the circular mean of the phase difference for the explicit observation condition is highest with 49.55°. The mean phase differences for the semi-implicit and implicit observation conditions are lower at 18.45° and 31.94°. Moreover, the mean resultant lengths of the three groups differ. The phase differences of the individuals in the explicit observation condition are most concentrated with $\bar{R}=0.77$. The phase differences differ more between the individuals in the semi-implicit and implicit observation conditions where the spread is larger at $\bar{R}$'s of 0.54 and 0.56, respectively.

Table 1 also shows a circular variance and standard deviation. The sample value, *V*_*m*_ for the circular variance is defined as $1-\bar{R}$. Its interpretation is exactly opposite to the interpretation of the mean resultant length. A variance of one means that a variable has a very large spread and a variance of 0 means that all data are concentrated at one point. Note that unlike a linear variance the circular variance is bounded between 0 and 1. A sample circular standard deviation, *v*, can also be computed (Fisher, 1995). This deviation runs from 0 to infinity where higher values indicate a larger dispersion.

We have seen that both the average phase difference and variances of the phase difference seem to be different for the three conditions in the motor resonance data. To test whether these differences in circular means also exist in the population, we can use a projected normal circular GLM. In the next section we will introduce this model and fit it to the motor resonance data.

## 4. A general linear model with a circular outcome

In this section we will introduce a projected normal circular regression model. Note that because it is a regression model we can also fit AN(C)OVA type models with it, we can thus refer to it as a projected normal (PN) circular GLM. The PN circular GLM falls within the embedding approach to circular data. The embedding approach is characterized by the fact that it takes an indirect approach to modeling circular data. Instead of directly defining a model on the circular outcome θ we use a mathematical trick that allows us to define a model in bivariate linear space. The results of the model in bivariate linear space can then be translated back to the circle. Next, we will outline the theoretical background to the PN circular GLM and the embedding approach. Subsequently we will continue to fit an ANOVA to the motor resonance data. At the end of this section we will shortly consider different methods for circular ANOVA.

### 4.1. The embedding approach to circular data

**Figure 5:**
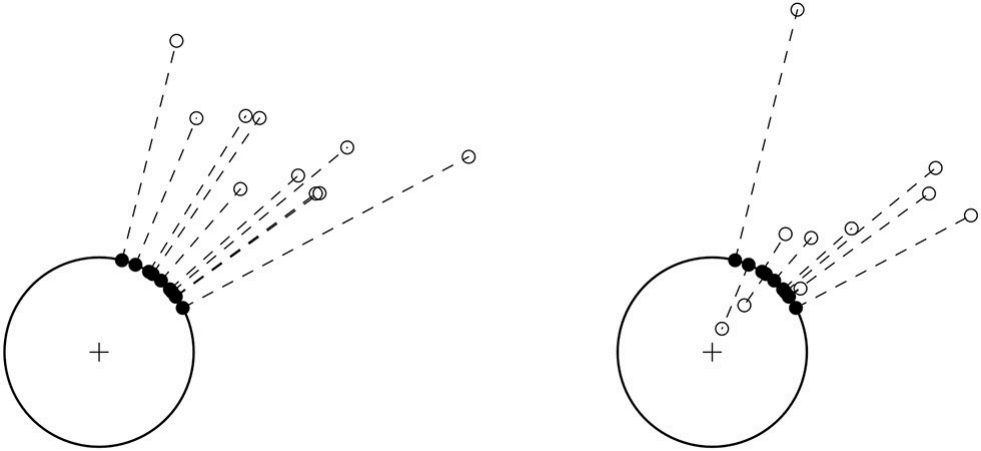
A set of circular datapoints (closed dots) connected to two sets of datapoints in bivariate space (open dots) that could have produced them.

In the previous section, at the computation of the circular mean, we have seen that a circular variable θ, e.g., the phase difference in the motor resonance data, can be expressed as a unit vector ***u*** composed of the sine and the cosine of an angle ***u*** = (cosθ, sinθ). If we translate this to bivariate real space the cosine is the x-component and the sine is the y-component of an angle. In the embedding approach we assume that ***u*** origins from a bivariate normal variable ***y***. This bivariate variable is not measured, and can thus be regarded as a latent variable. Figure 5 depicts the relation between ***u*** and ***y*** for a dataset with sample size *n =* 10. It shows that the circular datapoints could have originated from different sets of bivariate datapoints ***y***. Because the ***y*** are not observed we need special inference methods such as expectation maximization techniques in a frequentist approach or auxiliary variable techniques in a Bayesian approach to estimate a model. In this paper we will use a Bayesian approach. The reason to choose a Bayesian approach instead of a frequentist one is that it allows for the modeling of more complex data, e.g., there is no frequentist version of the circular mixed-effects model we will use in section 5. More details on the Bayesian approach can be found in Nuñez-Antonio et al. (2011) and Cremers et al. (2018).

From the assumption that ***y*** has a bivariate normal distribution it follows that θ has a projected normal distribution (Presnell et al., 1998). When fitting a model using the PN distribution we model the mean vector ***μ*** of the underlying bivariate data ***y***.

Because ***y*** is bivariate the mean vector ***μ*** has two components, denoted with the superscripts *I* and *II*. These superscripts therefore refer to the x and y axis of the Cartesian plane or the cosine and sine component in ***u***, respectively. In a multiple regression model this ***μ*** is specified as follows:

(1) $$\mu_{i}=\left(\begin{array}{c} \mu_{i}^{I}\\ \mu_{i}^{II} \end{array}\right)=\left(\begin{array}{c} {\left(\beta^{I}\right)}^{t}x_{i}^{I}\\ {\left(\beta^{II}\right)}^{t}x_{i}^{II} \end{array}\right),$$

where *i =* 1, …, *n*, ***x***_*i*_ is a vector of predictor values for individual *i* and each ***β*** is a vector with intercept and regression coefficients. The superscript *t* for $\beta_{0}^{I}$ and $\beta_{0}^{II}$ denotes that the transpose of these vectors is taken. To be able to estimate an intercept, the first component of ***x***_*i*_ equals 1. Note that the vectors ***x***_*i*_ are allowed to differ for the two components *I* and *II*. In the next section we will fit this type of model to the motor resonance data.

In terms of the interpretation of the circular effect of a variable the two component structure in (1) poses a problem. Note that we could rotate and shift the components (axes) in bivariate space such that for a categorical predictor the x-component points to the mean of the reference category and the beta weights of the y-component refer to a deviation from this reference mean. This way we could test whether the means of the groups differ in bivariate space. However, this still does not lead to means or effects that are interpretable on the circle. The two components do not necessarily have a useful interpretation for each type of circular data, e.g., we cannot talk of a 12 o'clock (sine component) and 3 o'clock axis (cosine component) in Figure 2. To be able to interpret effects on the circle we transform the effects on the two components to an effect on the circle. This transformation was introduced by Cremers et al. (2018) and will be applied in both example datasets.

### 4.2. Fitting an ANOVA model to the motor resonance data

In this section we will fit a circular ANOVA model to the motor resonance data using the PN circular GLM from the package bpnreg. Note that the model from this package is in fact a regression model that we can speficy in such a way that it is mathematically equivalent to an ANOVA. First we will give the details on this model and subsequently we will discuss and interpret the results.

#### 4.2.1. Fitting the model

To investigate the effect of condition on the phase difference we specify the prediction equation for the mean vector in the PN circular GLM as follows:

(2) $$\mu_{i}=\left(\begin{array}{c} \mu_{i}^{I}\\ \mu_{i}^{II} \end{array}\right)=\left(\begin{array}{c} \beta_{0}^{I}+\beta_{1}^{I}\text{semi}{\text{.implicit}}_{i}+\beta_{2}^{I}{\text{implicit}}_{i}\\ \beta_{0}^{II}+\beta_{1}^{II}\text{semi}{\text{.implicit}}_{i}+\beta_{2}^{II}{\text{implicit}}_{i} \end{array}\right),$$

where the variables semi.implicit and implicit are dummy variables indicating condition membership, $\beta_{0}^{I}$ and $\beta_{0}^{II}$ are the intercepts and $\beta_{1}^{I}$, $\beta_{2}^{I}$, $\beta_{1}^{II}$ and $\beta_{2}^{II}$ are the regression coefficients of the model. Note that in this model we take the explicit observation condition as the reference condition. When we translate this to the ANOVA context, the intercepts, $\beta_{0}^{I}$ and $\beta_{0}^{II}$, represent the mean for the explicit condition, $\beta_{0}^{I}+\beta_{1}^{I}$ and $\beta_{0}^{II}+\beta_{1}^{II}$ are expressions for the mean of the semi-implicit condition and $\beta_{0}^{I}+\beta_{2}^{I}$ and $\beta_{0}^{II}+\beta_{2}^{II}$ are expressions for the mean of the implicit condition.

We use the package bpnreg to fit the model. Because this is a Bayesian method we have to specify some parameters for the Markov Chain Monte Carlo (MCMC) sampler that estimates the parameters of the model. MCMC methods are iterative and we thus need to specify the number of iterations that we want to run. We choose a relatively high number of the output iterations (its = 10000) to make sure that the sampler converges and that we don't need to run it again in case it did not. We choose the burn-in period to consist of 100 iterations (burn = 100). This means that we throw away the first 100 iterations to make sure that the iterations we keep are those at which the sampler has reached its equilibrium. We also choose the lag, that is how many iterations we want to keep, in this case every third iteration (n.lag = 3). We set a lag to prevent possible auto-correlation between the parameter estimates. In the next section we will elaborate further on how to check convergence and choose these MCMC parameters wisely. But first, we fit the model:

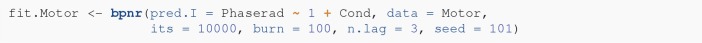



#### 4.2.2. Convergence

**Figure 6:**
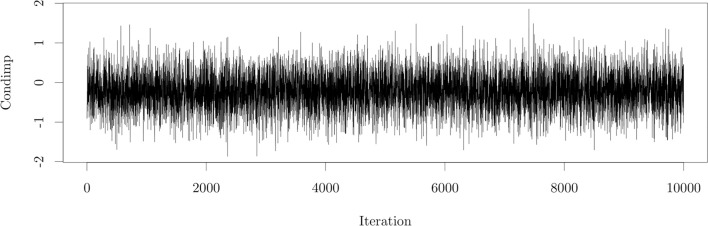
A traceplot showing convergence of the parameter $\beta_{2}^{I}$ of the model fit to the motor resonance data.

In a Bayesian model that uses MCMC sampling for estimation we always have to assess convergence of the MCMC chain for all parameters in the model. A traceplot is one way to assess the convergence of a parameter. As an illustration we only show the traceplot for the MCMC chains for one of the parameters of the model in Figure 6.

From the traceplot in Figure 6 we may conclude that the MCMC chain converged within 10,000 iterations and a burn-in of 100. In general if the traceplot shows proper convergence there are no flat parts, where the chain stays in the same state for too long, or parts with too many steps in one direction. We want to see a pattern in which the chain moves from below a certain equilibrium to above and vice versa in just a few iterations. The traceplot will then look like a so-called “fat-caterpillar” meaning that the chain has reached an equilibrium around a particular value and has thus converged. In case an MCMC chain does not converge we could add more iterations, a larger n.lag or more burn-in iterations. We can also evaluate other convergence diagnostics. The focus of this paper however does not lie on Bayesian data analysis and therefore we refer to other works (e.g., Gelman et al., 2014), for more information on assessing convergence.

#### 4.2.3. Results

To answer the question whether the phase differences in the three conditions of the motor resonance data differ we investigate their circular means. To do so we use methods from Cremers et al. (2018) to transform the results from the two components of ***μ*** to the circle. Note that investigating the regression coefficients on the two bivariate components separately might lead to wrong conclusions about the effect on the circle, the phase difference. This is due to the fact that even though there is an effect of a variable on each of the two components this does not mean that we can also see an effect on the circle. For a more detailed explanation we refer to Cremers et al. (2018).

Because we use a Bayesian method we get the posterior distributions of the three circular means. Philosophically, in Bayesian statistics each parameter is said to have its own distribution. The posterior distribution is the result of the prior knowledge we have about a parameter before conducting a study, formalized as a “prior” distribution (in this paper we choose non-informative priors for the parameters) and the information that lies in the data obtained from a study, formalized as the likelihood. The fact that we obtain the distribution of a parameter is convenient for inference purposes since this means that we do not just have a point estimate of a parameter (the mean or mode of the posterior distribution) but we also automatically get an uncertainty estimates (the standard deviation of the posterior distribution). For more background on Bayesian statistics (see e.g., Gelman et al., 2014).

Summary statistics for the posterior distributions of the circular means for each condition are shown in Table 2. This table shows the posterior mean, mode, standard deviation (sd) and the lower and upper bound of the 95% highest posterior density interval (HPD). The standard deviation of a posterior is an estimate for the standard error of the parameter. The HPD interval is the smallest interval in which 95% of the posterior distribution is located. In terms of interpretation, it is different from a frequentist confidence interval since HPD intervals allow for probability statements. For example, if the 95% HPD interval for a parameter μ runs from 2 to 4 we can say that the probability that μ lies between 2 and 4 is 0.95.

**Table 2:** Posterior estimates of the circular means of the phase difference for the three conditions of the motor resonance data.

**Condition**	**Mode**	**Mean**	**sd**	**LB HPD**	**UB HPD**
Explicit	42.70°	45.56°	11.67°	22.26°	67.99°
Semi-implicit	21.08°	19.40°	18.36°	–18.27°	55.22°
Implicit	37.22°	33.47°	17.77°	–2.25°	68.22°

HPD intervals can also be used to test whether a parameter is different from a certain value or whether two parameter estimates are different. In Table 2 we see that the HPD intervals of the circular means for the three conditions in the motor resonance data overlap. The circular mean of the phase difference is estimated at 47.70° (22.26°; 67.99°) for the explicit condition, 37.22° (–2.25°; 68.22°) for the implicit condition and 21.08° (–18.27°; 55.22°) for the semi-implicit condition. Because the HPD intervals of these estimates overlap, we conclude that there is not enough evidence to reject the null hypothesis that the circular means for the three conditions do not differ and that there is no effect of condition on the average phase difference. Note that the fact that no difference was found may be due a lack of power caused by the relatively small sample size (*N* = 14).

In addition to testing whether the circular means of the three conditions are different, the circular ANOVA also allows us to test whether there is an effect of condition on the circular variances of the phase differences. Table 3 shows summary statistics for the posterior distributions of the circular variance for each condition. As expected the estimated circular variance for the explicit condition is lowest. However, the variances of the three groups do not significantly differ; their HPD intervals overlap. We thus conclude that there is no evidence for an effect of condition on the variance of the phase difference. Note that a function to compute these variances has not yet been implemented in version 1.0.0 of bpnreg. It is however possible to get the MCMC estimates from the fit object and subsequently use Equation 3 from Kendall (1974) on the estimated mean vector for each of the groups to compute the variances.

**Table 3:** Posterior estimates of the circular variances of the phase difference for the three conditions of the motor resonance data.

**Condition**	**Mode**	**Mean**	**sd**	**LB HPD**	**UB HPD**
Explicit	0.21	0.26	0.09	0.09	0.45
Semi-implicit	0.37	0.44	0.13	0.19	0.36
Implicit	0.36	0.42	0.13	0.18	0.68

### 4.3. Other approaches to circular ANOVA

In the previous section we have tested whether the average phase differences of the three conditions of the motor resonance data differ in the population using a Bayesian PN circular GLM. We can also do this using a frequentist ANOVA for circular data that tests the hypothesis *H*_0_:μ_*explicit*_ = *μ*_*semi*−*implicit*_ = *μ*_*implicit*_. One of such tests is the Watson-Williams test. This test can be performed using the function watson.williams.test in the circular package and is similar to an ANOVA for linear data interpretation-wise. Note that the Watson-Williams test falls within a different approach to modeling circular data, the intrinsic approach. In this approach we directly model the circular data instead of making use of a mathematical trick that allows us to model the data in bivaraite space and then translate the results back to the circle. For simple models, such as the ANOVA, this modeling approach works fine. However, for more complex data structures we have a much larger choice of models in the embedding approach. For example, a disadvantage of the Watson-Williams test is that it does not allow for the addition of covariates and thus cannot estimate AN(C)OVA models. The PN circular GLM does allow for the addition of covariates.

As in ANOVA models for linear data, we have to meet a set of assumptions for this test to be valid. Firstly, in the Watson-Williams test the samples from the different conditions are assumed to be von-Mises distributed. Like the projected normal distribution this is a distribution for circular data. It is unimodal with mean μ and concentration κ. Secondly, the samples are assumed to have the same circular variance. This assumption of homogeneity of variance is tested within the watson.williams.test function itself. For the motor resonance data this assumption was met. The assumption of von-Misesness can be tested using e.g., the Watson's goodness of fit test for the von Mises distribution. If we perform this test on the phase differences of the three subgroups we conclude that only the phase differences from the semi.implicit and the implicit condition are von-Mises distributed (*H*_0_ is not rejected). This means that it is not completely valid to perform the Watson-Williams test on the motor resonance data.

For educational purposes however we do decide to conduct this test. Similar to the projected normal circular GLM we conclude from this test that the average phase differences of the three conditions are not significantly different: [*F*_(2, 39)_ = 1.02, *p* > 0.05].

## 5. Mixed-effects models with a circular outcome

An advantage of employing the embedding approach to circular data over the intrinsic approach is that it is easier to model more complex data, e.g., repeated measures data, since we can “borrow” methods from the bivariate linear context. In this section we will introduce such a method: the circular mixed-effects model. We will first introduce a new dataset, the cognitive maps data, and give descriptive statistics. Then, we will shortly outline the theoretical background to the mixed-effects model and fit it to the cognitive maps data.

### 5.1. The cognitive maps data

The cognitive maps data is a subset of data from a study by Warren et al. (2017) on the geometry of humans' knowledge of navigation space. In their study Warren et al. (2017) amongst others conduct an experiment in which a total of 20 participants used virtual reality headsets to navigate through one of two virtual mazes. The navigation task consisted of walking from a start object to a target object. In a training phase they had learned to navigate between different pairs of start and target objects in one of two versions of the maze. The number of trials each participant completed in this training phase was recorded. In the test phase of the experiment participants first walked to a start object. When they had reached this object the maze dissappeared and only a “textured groundplane” of the maze remained visible. The participants then turned toward the location of the target object that they had remembered during the training phase and started to walk toward the target. The angular difference between the initial walking direction of a participant from the start object and the location of the target object, that is, the angular error, was recorded as an outcome variable in the experiment.

The type of maze is a between-subjects factor, participants either had to navigate through a “Euclidean” maze or a “non-Euclidean” maze. The Euclidean maze is the standard maze and is a maze just as we know it in the real world. The other version of the maze, the non-Euclidean maze, has exactly the same layout as the standard maze but it has virtual features that do not exist in reality. It namely contains wormholes by which participants can be “teleported” from one place in the maze to another.

In the test phase of the experiment all participants had to complete 8 trials. In each of these trials participants had to walk to a specific target object. A within-subjects factor is the type of target object. Pairs of start and target objects were of two types: probe and standard. The probe objects were located near the entrance and exit of a wormhole in the non-Euclidean maze whereas the standard objects were located at some distance from the wormholes. For each of these two types of objects participants had to find 4 different targets resulting in a total of 8 trials per participant.

For this experiment we could be interested in the question whether the participants in the non-Euclidean maze make use of the wormholes when navigating to the target objects and whether this is true for both the probe and standard target objects. Due to the design of the mazes the expected angular error was larger if a participant used the wormhole to walk to the target object in the non-Euclidean maze. We can thus use the angular error, our outcome variable, to differentiate between participants that used the wormhole and those that took another path to the target object. Additionally we can control for the amount of trials that a participant completed in the training phase.

### 5.2. Descriptive statistics

The cognitive maps data is incorporated in the package bpnreg as the dataframe Maps. This dataframe has 160 rows; there are 20 subjects that each completed 8 trials. The data contains an index variable for the subject Subject (*N* = 20) and trial number Trial.no (*n* = 8). It also includes variables indicating the type of maze Maze, a between-subjects factor, and type of trial Trial.type, a within-subjects factor. The variable Learn indicates the amount of learning trials completed. L.c is a centered version of this variable. The angular error is contained in the variables Error and Error.rad in degrees and radians, respectively. Descriptives for this data are shown in Table 4. Note that we averaged over subjects and the trials of each type. The circular mean of the angular error for the standard trials in the Euclidean maze is thus an average over 10 participants and 4 trials. We see that the average angular errors, $\hat{\theta }$, for the non-Euclidean maze deviate more from 0° (direction of the target object) than for the Euclidean maze.

**Table 4:** Descriptives for the cognitive maps data with mean direction ($\bar{\theta }$) and mean resultant length ($\bar{R}$) of the angular error for each condition.

	**Maze**	**Trial.type**	**$\bar{\theta }$**	**$\bar{R}$**
Angular error	Euclidean	Standard	–4.91°	0.89
		Probe	4.46°	0.92
	non-Euclidean	Standard	–17.59°	0.78
		Probe	37.34°	0.93

### 5.3. Fitting a mixed-effects model to the cognitive maps data

In this section we will first introduce a circular mixed effects model and fit this model to the cognitive maps data. Next we discuss the output produced by the bpnreg package. We will discuss the interpretation of fixed and random effects and model fit.

#### 5.3.1. The embedding approach for mixed-effects models

The circular mixed-effects model from the package bpnreg is also based on the embedding approach to circular data. The basic idea behind this approach is the same as outlined before. In a real dataset we have a set of outcome vectors ***u***_*ij*_, one for each measurement *j* within a higher level observation *i*. We however estimate a model to the underlying bivariate data ***y***_*ij*_. The Bayesian method used in the package bpnreg for estimating circular mixed-effects models is outlined in Nuñez-Antonio and Gutiérrez-Peña (2014).

For the cognitive maps data with *i =* 1, …, 20 individuals and *j =* 1, …, 8 measurements per individual we fit a mixed-effects model to investigate the influence of the type of Maze, type of trial and amount of learning trials on the angular error. The prediction for the mean vector in this model, ***μ***, is specified as follows:

(3) $$\mu_{ij}=\left(\begin{array}{c} \mu_{ij}^{I}\\ \mu_{ij}^{II} \end{array}\right)=\left(\begin{array}{c} \beta_{0}^{I}+\beta_{1}^{I}{\text{Maze}}_{i}+\beta_{2}^{I}\text{Trial}{\text{.type}}_{ij}+\beta_{3}^{I}\text{L}{\text{.c}}_{i}+b_{0i}^{I}\;\\ \beta_{0}^{II}+\beta_{1}^{II}{\text{Maze}}_{i}+\beta_{2}^{II}\text{Trial}{\text{.type}}_{ij}+\beta_{3}^{II}\text{L}{\text{.c}}_{i}+b_{0i}^{II} \end{array}\right),$$

where the variables Maze and Trial.type are dummy variables, $\beta_{0}^{I}$ and $\beta_{0}^{II}$ are the fixed intercepts, $b_{0i}^{I}$ and $b_{0i}^{II}$ are the random intercepts and $\beta_{1}^{I}$, $\beta_{2}^{I}$, $\beta_{3}^{I}$, $\beta_{1}^{II}$, $\beta_{2}^{II}$ and $\beta_{3}^{II}$ are the fixed regression coefficients of the model. Note that in this model we take the Euclidean maze and standard trials as reference conditions.

The interpretation problems caused by the two component structure in (3) is of a similar nature as the one in the GLM model. Cremers et al. (Submitted) introduce new tools that solve the interpretation of circular effects in PN mixed-effects models. In this tutorial we will also use these tools.

#### 5.3.2. Fitting the model

To fit the model in (3) we use the bpnme() function from the package bpnreg. We also need to specify some parameters for the MCMC sampler that estimates the model. We specify the output iterations (10,000), the amount of burn-in (1000) and how many iterations we want to keep (n.lag = 3). Convergence was checked in the same manner as for the ANOVA model in the previous section and was reached using the settings for the MCMC algorithm we just specified.

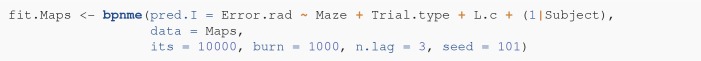



Note that the syntax for the model specification in this function is similar to that of the package lme4 for fitting (non-circular) mixed-effects models.

#### 5.3.3. Fixed effects

Next we investigate the coefficients of the fixed effects for this model. First we show results for the categorical variables type of maze (Maze) and type of trial (Trial.type).

Table 5 shows summary statistics of the posterior of the average angular error for each of the categories. Note that because there is a continuous predictor in the model the posterior estimates represent a marginal effect, they are the effect for an individual with a 0 score on the continuous predictor L.c. Because we centered this predictor this means that this is the effect for an individual that has completed an average number of training trials.

**Table 5:** Posterior estimates of the circular mean of the angular error for each condition.

	**Maze**	**Trial.type**	**Mode**	**Mean**	**Sd**	**LB HPD**	**UB HPD**
Angular error	Euclidean	standard	–12.97°	–13.48°	3.9°	–21.42°	–6.06°
		Probe	11.38°	11.78°	3.29°	5.26°	18.30°
	non-Euclidean	Standard	–1.42°	–2.04°	6.68°	–15.75°	10.49°
		Probe	31.04°	30.37°	4.31°	22.03°	38.92°

By looking at the 95% HPD intervals of the angular errors in Table 5 we can test whether the type of maze and type of trial on average has an influence on the angular error and thus whether participants make use of the wormhole. For the standard trials we see that the HPD intervals of the angular error in the Euclidean and non-Euclidean overlaps and that thus the angular error is not different. This means that in the standard trials the participants on average did not make use of the wormholes in the non-Euclidean maze. For the probe trials however, the HPD intervals of the Euclidean and non-Euclidean do not overlap and thus the angular error is different. This means that in the probe trials, the participants on average did make use of the wormholes in the non-Euclidean maze.

For the continuous variable L.c we get a set of parameters, *b*_*c*_, *SAM* and *AS*, describing its effect on the circle. How these parameters are computed is described in Cremers et al. (2018) and Cremers et al. (Submitted) In this paper we will only focus on how to interpret them.

**Figure 7:**
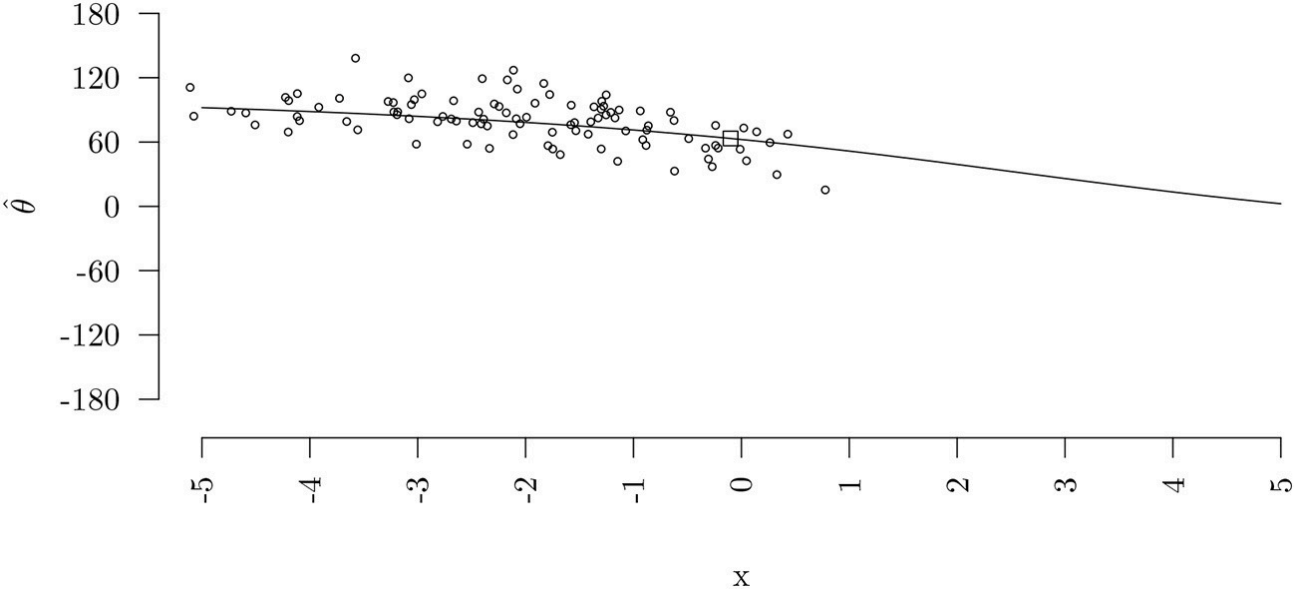
Predicted circular regression line for the relation between a linear predictor *x* and a predicted circular outcome θ together with the original datapoints. The square indicates the inflection point of the regression line.

In Figure 7 a circular regression line for the effect of a predictor *x* on the circular outcome is shown. Because the outcome variable is measured on a circular scale, the slope of this line (the effect of *x*) is not constant but different for different *x* values. The regression line can be described using the three circular coefficients *b*_*c*_, *SAM* and *AS*. The coefficient *b*_*c*_ represents the slope of the circular regression line at the inflection point (the square in Figure 7). However, this may not be a representative effect for each dataset as the inflection point can lie in the extremes of the data (as in Figure 7) or even completely outside the range of the predictor *x*. Therefore, two additional circular coefficients were developed by Cremers et al. (2018), the slope at the mean *SAM* and the average slope *AS*. The coefficient *SAM* represents the slope of the circular regression line at the average of the predictor ($\bar{x}$) and the coefficients *AS* represents the average slope over all values of *x*.

For the effect of L.c on the angular error in the cognitive maps data, the HPD intervals for all three circular coefficients, *b*_*c*_, *SAM* and *AS* include 0 (see Table 6). Thus, we do not find evidence that at the inflection point, at the average predictor value and on average the number of training trials (L.c) influences the angular error. Note that there not being evidence for influence is a good thing, since it indicates that the training phase of the experiment worked to get all participants at the same level. We do however need to be wary of making all to strong conclusions, we are essentially trying to find evidence for a null-hypothesis with a small sample (*n* = 20). If the sample had been larger we would have had more power to reject the hypothesis, possibly resulting in the opposite conclusion. For educational purposes we continue to give the interpretation of the coefficients. The *SAM* is interpreted as follows: at the average L.c, for a 1 unit increase in L.c the angular error increases with 0.58 degrees. The *AS* can be interpreted as: on average, for a 1 unit increase in L.c the angular error decreases with 0.63 degrees on average. The *b*_*c*_ can be interpreted as: at the inflection point, for a 1 unit increase in L.c the angular error decreases with 0.89 degrees.

**Table 6:** Posterior estimates of the coefficients of the effect of L.c on the angular error.

**Coefficient**	**Mode**	**Mean**	**Sd**	**LB HPD**	**UB HPD**
*b*_*c*_	–0.89°	–0.21°	1.73°	–2.84°	2.55°
*SAM*	0.58°	–0.84°	90.80°	–11.51°	12.73°
*AS*	–0.63°	–1.11°	92.44°	–13.17°	13.14°

#### 5.3.4. Random effects

In mixed-effects models we are also interested in evaluating the variance of the random effects. In the model for the cognitive maps data we included a random intercept. This means that we estimate a separate intercept for each participant. How to compute random effect variances on the circle is outlined in Cremers et al. (Submitted) For the cognitive maps data the posterior mode of the intercept variance on the circle is estimated at 3.5*10^−5^ and its HPD interval is (4.2*10^−6^; 1.4*10^−3^). This variance is very low which means that the participants do not differ a lot in their individual intercept estimates. Note that this is not necessarily problematic. In some cases we are not interested in the variances of the random effects but simply want to fit a mixed-effects model because we have within factors, such as Trial.type, that cannot be properly incorporated in a standard regression model.

#### 5.3.5. Model comparison

When fitting mixed-effects (or multilevel) models we often fit a set of nested models to our data and follow a model building strategy (Hox, 2002). We do this in case we have no specific model in mind that we want to test and want to explore the individual contributions of variables or groups of variables to the model. Such a model building strategy can be done top-down, starting with the most complex model, or bottom-up, starting with the simplest model. Here we use a bottom-up strategy and start with the so called intercept-only model, a model containing only a fixed and random intercept:

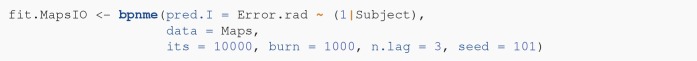



We then update this model with fixed effects for the predictors at the lowest level (within-subjects factors), in this case Trial.type. We do this to check whether the set of predictors improved the fit of the model and can explain a part of the random intercept variance from the intercept-only model.

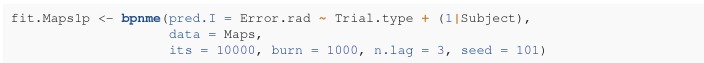



We then add fixed effects for the predictors at the higher level (between-subjects factors), in this case Maze and L.c. Again we do this to check whether they improve the fit of the model and whether they can explain a part of the random intercept variance.

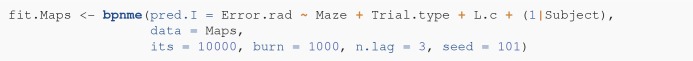



Because we have already seen that the effect of L.c was not different from 0 we also fit the model with only the Maze and Trial.type predictors.

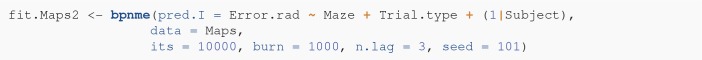



Additional steps, such as adding random slopes for first level predictors and cross-level interactions, can be taken. In this paper we will however restrict the analysis to the previous three models.

**Model Fit**

To assess the fit of the models we look at 4 different model fit criteria: two version of the deviance information criterion (DIC and DIC_*alt*_) and two versions of the Watanabe-Akaike information criterion (WAIC_1_ and WAIC_2_). We choose these four criteria because they are specifically useful in Bayesian models where MCMC methods have been used to estimate the parameters. All four criteria have a fit part consisting of a measure based on the loglikelihood and include a penalty in the form of an effective number of parameters. For all criteria lower values indicate better fit. Gelman et al. (2014) describes how to compute these criteria. Table 7 shows the results of these criteria for four different models.

**Table 7:** Model fit criteria for several models fit to the cognitive maps data.

**Criterion**	**Intercept-only**	**Trial.type**	**Trial.type+ Maze**	**Trial.type+ Maze+ L.c**
DIC	304.61	267.91	253.97	257.94
DIC_*alt*_	324.33	286.97	257.14	260.78
WAIC_1_	308.41	271.61	255.00	258.41
WAIC_2_	308.43	271.77	255.40	259.02

In the results for the example we see that the fit improves in all 4 model diagnostics for each model except for the last one. This means that the predictor Trial.type improves the fit of the model over the intercept-only model and that the predictors Maze and Trial.type together improve the fit of the model over the model with only the Trial.type predictor. Because the variable L.c had no effect it is as expected that this predictor does not improve the fit of the most right model over the model with the Maze and Trial.type predictors. We conclude that the model with the predictors Trial.type and Maze fits best.

**Explained Variance**

Apart from information about whether adding predictors improves the fit of the model we are also interested in whether these predictors explain a part of the random effect variances. For the cognitive maps data we are interested in whether the Maze and Trial.type predictors explain a part of the variance in individual intercepts. To assess this we compare the posterior estimates of the circular random intercept for the intercept-only model and the model with the Maze and Trial.type predictors.

The posterior mode of the intercept variance in the intercept-only model equals 6.61 ∗ 10^−5^(8.20 ∗ 10^−6^; 3.62 ∗ 10^−3^). This means that there is almost no random intercept variance. The posterior mode of the circular variance is very close to 0. This also means that there is hardly any intercept variance that the Maze and Trial.type predictors can explain. For illustrative purposes however we continue to assess the intercept variance in the model with the Maze and Trial.type predictors. The posterior mode of the intercept variance in the model with Maze and Trial.type equals 3.25 ∗ 10^−5^(4.40 ∗ 10^−6^; 1.59 ∗ 10^−3^). As expected, there is hardly any change in estimates for the variance in the model with Maze and Trial.type compared to the intercept-only model. Furthermore, their HPD intervals have a very large overlap. We thus conclude that the variables Maze and Trial.type did not explain any variance in the random intercepts.

## 6. Concluding remarks

In this paper we have given a tutorial for researchers in cognitive psychology on how to analyse circular data using the package bpnreg. We have covered data inspection in section 3, the fitting of a Bayesian circular GLM in section 4 and the fitting of a Bayesain mixed-effects model in section 5. We have also given a short introduction into the theoretical background of these models in sections 4.1 and 5.3.1.

Apart from the embedding approach to circular data, as used in this tutorial, there are two other approaches to the analysis of circular data. In the wrapping approach the data on the circle is assumed to have originated from wrapping a univariate distribution on the real line onto the circle. In the intrinsic approach distributions, such as the von Mises distribution, are directly defined on the circle. For both approaches models have been described in the literature (Fisher and Lee, 1992; Gill and Hangartner, 2010; Ravindran and Ghosh, 2011; Lagona, 2016; Mulder and Klugkist, 2017). The regression model using the intrinsic approach from Fisher and Lee (1992) is a frequentist method and is implemented in the package circular and the circular general linear model from Mulder and Klugkist (2017) is a Bayesian method which is implemented in the package circglmmbayes. For neither approach however a detailed tutorial describing how to analyze circular data using the functions from their package has been written thus far. Furthermore, the PN approach to circular modeling has the additional advantage that it is relatively easy to fit more complex models, e.g., the mixed-effects model in this tutorial.

## Author contributions

The idea for this paper was conceived by JC with feedback from IK. JC performed the data analysis and developed the software package (bpnreg) to execute them. Methodology from the software package (bpnreg) was developed by JC with contributions from IK. Manuscript textual content, formatting, and figures were produced by JC. IK contributed to manuscript revision, read, and approved the submitted version.

### Conflict of interest statement

The authors declare that the research was conducted in the absence of any commercial or financial relationships that could be construed as a potential conflict of interest.
